# Characterization of flavor substances in cooking and seasoned cooking brown seaweeds by GC-IMS and E-nose

**DOI:** 10.1016/j.fochx.2024.101325

**Published:** 2024-03-26

**Authors:** Shan Jiang, Pengfei Jiang, Dingding Feng, Meiran Jin, Hang Qi

**Affiliations:** SKL of Marine Food Processing & Safety Control, National Engineering Research Center of Seafood, Collaborative Innovation Center of Seafood Deep Processing, School of Food Science and Technology, Dalian Polytechnic University, Dalian 116034, China

**Keywords:** Cooking, Seasoned cooking, Volatile flavor components, *Laminaria japonica*, *Undaria pinnatifida*

## Abstract

The flavor of algae was one of the key factors for consumer acceptance. The objective of this study was to investigate the characteristic volatile compounds in cooking and seasoned cooking edible brown seaweeds (*Undaria pinnatifida* and *Laminaria japonica*). The gas chromatography-ion mobility spectrometry (GC-IMS) and electronic nose (E-nose) analysis showed that baking resulted in significant difference in flavor of brown seaweeds. However, the overall effect of cooking was not as significant as that of the seasoning solution treatment. Additionally, brown seaweeds treated with the seasoning solution were more acceptable. *Undaria pinnatifida* was found to contain 72 volatile flavor compounds, while *Laminaria japonica* had a total of 70. This study proved the applicability of GC-IMS combined with E-nose technology to detect the changes of volatile components of brown seaweeds after processing, providing beneficial knowledge and basic theory for the deep processing of brown seaweeds.

## Highlights

• GC-IMS and E-nose assess the flavor characteristics of brown algae.
• 72 characteristic volatile compounds were detected in *Undaria pinnatifida*, and 70 in *Laminaria japonica*.
• The aroma characteristics of edible brown algae treated with seasoning solution were significantly enhanced.

## Graphical abstract

**Figure:**
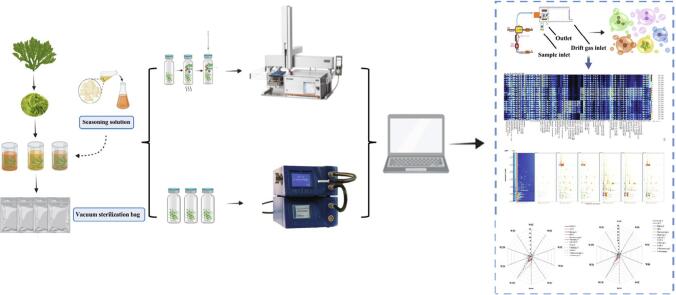

## 1 Introduction

Algae is a kind of heterogeneous plant group with a long history without root and leaf system, which is mainly divided into microalgae and macroalgae (seaweeds) (Gupta & Abu-Ghannam, 2011). The bioactive substances found in seaweeds include lipids, proteins, polysaccharides, phenols, and pigments. They have biological activities that include those that are antibacterial, anti-cancer, anti-inflammatory, anti-diabetes, and anti-obesity (Sapatinha et al., 2022). In addition, seaweeds' secondary metabolites are a good source of numerous vitamins and minerals that the body need, such as vitamin A, C, D and E, niacin and folic acid, calcium, sodium, potassium, etc. (Pereira, 2005). As a result, seaweeds have long been a healthy food source and part of the diet of Asians (Wijesinghe & Jeon, 2012). They are also attracting attention as a valuable source of functional ingredients in the nutritional, medical and cosmetic industries (Park & Lee, 2021). Based on their chemical makeup, seaweeds are divided into green, brown, and red seaweed, with brown seaweed being the most popular for human consumption (Jiang et al., 2021).

*Undaria pinnatifida* (*U. pinnatifida*) and *Laminaria japonica* (*L. japonica*) are the most commonly consumed edible brown seaweed. For dietary or medical purposes, they are seen as a potential sustainable marine resource. They have potential health advantages since they are rich source of bioactive substances, including dietary fiber, polyphenols, fatty acids and carotenoids (Yue et al., 2022). For example, *U. pinnatifida* is widely regarded as a long-standing “longevity food” or “sea of vegetables” in many Western countries (Martinez-Villaluenga et al., 2018). The classic ancient Chinese text, Materia Medica, documented its diuretic and laxative properties. It has been used throughout Chinese history to treat conditions such as hyperthyroidism, oedema and haemorrhoids (Zeng et al., 2022). Previous studies have revealed that *L. japonica* has beneficial health effects such as antioxidant, immunomodulatory and hypolipidemic activities (Guo et al., 2014, Huang et al., 2010, Zha et al., 2015). In addition to containing a wide range of biologically active substances, *U. pinnatifida* and *L. japonica* have a special marine aroma. Li et al. (2023) found that some volatile compounds in seaweed have a significant impact on its flavor. These volatile compounds produce different odors, such as “seafood”, “licorice”, “spices”, “honey”, and “fruit”, which can affect consumer preference for seaweed food. Although a few studies have reported the volatile component composition of seaweeds, the flavor composition of seaweeds treated with different cooking techniques and seasoning liquids remains unclear (Lu, Yosemoto, Takayama, Satomi, & Akakabe, 2018).

The role of seasoning in the food industry, catering industry and domestic cooking is becoming more and more important. It is common for people to add seasonings to vegetables when cooking and processing them to make them more palatable. Studies have shown that improving the palatability and flavor of vegetables or vegetable dishes increases the acceptance of vegetables (Manero et al., 2017). Seasonings can mask or eliminate some of the undesirable flavors, enhance the color of food, and improve the taste and texture or appearance of vegetables, thus directly affecting people's enjoyment of the dish and increasing their appetite. In addition, some seasonings have their own nutritional elements, which are beneficial to human health when used in moderation. Therefore, seasoning plays an important part in the cooking of vegetables. For example, a study of children by Fisher et al. (2012). found that seasoning sauces affect vegetable intake. Manero's study found that seasoned broccoli and string beans were more popular with consumers than the unseasoned versions (Manero et al., 2017).

Volatile flavor compounds have a significant role in the flavor of food. They play a crucial role in the assessment of food products, as they are a key determinant of consumer interest and acceptance (C. Li, Al-Dalali, Wang, Xu, & Zhou, 2022). Different cooking methods improved the organoleptic and textural characteristics of brown seaweeds. For example, microwave-treated *Undaria pinnatifida* retained color, polysaccharides and total phenolic content better (Jiang et al., 2021). Cooking methods directly affect the nutritional properties and final composition of the food, while volatile flavor substances released during cooking have a significant effect on the taste and aroma of brown seaweeds. Different seasonings and cooking techniques will result in a variety of food flavors that may change greatly from raw ingredients. In recent years, instrumental analytical techniques have been developed to identify volatile flavor components in food. This enables a better understanding of the relationship between the chemical composition of food flavors and human senses when combined with subjective sensory assessments. It provides a more comprehensive and objective basis for people to evaluate food flavors (Wang, Chen, & Sun, 2020). Gas chromatography-ion mobility spectrometry (GC-IMS) is a very common instrumental analytical technique for the determination of volatile flavor compounds in foods. It is a new gas phase separation technique combining ion mobility spectrometry with gas chromatography. It has a fast detection time, no pre-treatment and stable detection results. Simultaneously, it overcomes the disadvantages of poor resolution of ion mobility spectrometry, and has been widely used in the analysis of flavor substances in food (Liu et al., 2021). For example, Guo et al., 2021, Guo et al., 2021 used CG-IMS to identify the aroma characteristics of fresh tea leaves and oolong tea. For the analysis of food flavors, electronic nose (E-nose) is also frequently used. The E-nose is a technology used for detecting aromas, using an array of gas sensors and pattern recognition technology for olfactory recognition. It has the advantages of low cost and high degree of automation, providing the overall information of volatile compounds and flavor for the detected samples. However, it cannot provide a detailed analysis of the sample (Yang et al., 2022). The GC-IMS and E-nose technologies may create a fingerprint and radar spectrum of volatile organic chemicals, allowing for direct sample comparison. The combination of the two technologies provides a reliable technical basis for the comprehensive analysis of food flavors.

In this study, we used the sensitivity and rapidity of GC-IMS detection combined with E-nose to analyze the effects of seasoning solution and different cooking techniques on the flavor compounds in *U. pinnatifida* and *L. japonica*, and to establish the corresponding flavor fingerprints and E-nose radar maps. This study aimed to fill the current research gap by detecting and analyzing flavor compounds in brown seaweeds (*U. pinnatifida* and *L. japonica*) treated with seasoning and different cooking methods through GC-IMS coupled with E-nose. And there were few relevant reports. Therefore, it provides a reference for the subsequent processing of edible brown seaweeds for aroma characterization and quality control, and provides a basis for the study of the flavor characteristics of edible brown seaweeds.

## 2 Materials and methods

### 2.1 Materials and chemicals

Dalian Aquaculture Group Co., Ltd. (Dalian, China) supplied the salted *U. pinnatifida*. The *L. japonica* was purchased by Shanghai Sanyuan Industrial Co., Ltd. (Shanghai, China). Food grade calcium chloride was obtained from Jiangsu Kolod Food Ingredients Co., Ltd. (Jiangsu, China). Edible salt was provided from China Salt Dongxing Salt & Chemical Co., Ltd. (Anhui, China). White granulated sugar was bought from Sugarman (Guangzhou, China). White vinegar was provided by Haday (Foshan, China). Mature vinegar was from Shanxi Shuita Vinegar Industry Co., Ltd. (Shanxi, China). The soy sauce was sourced from Heshan City Donggu Flavouring & Food Co., Ltd. (Heshan, China). Monosodium Glutamate was acquired from Shenyang Hongmei Food Co., Ltd. (Shenyang, China). 2-butanone, 2-pentanone, 2-hexanone, 2-heptanone, 2-octone, and 2-nonone (all analytical pure) were purchased from Shanghai Aladdin Biochemical Technology Co., Ltd. (Shanghai, China).

### 2.2 Sample preparation

#### 2.2.1 Pretreatment of *U. pinnatifida* and *L. japonica*

In order to remove the salt on the surface of salted *U. pinnatifida*, it was washed three to four times with deionized water. Then immersed it in water until it reached the expanded state. After removing the excess water, cut the *U. pinnatifida* into 6 cm long and 0.5 cm wide samples.

*L. japonica* was soaked in water for 30 min and then taken out. The excess water on the surface was removed and cut into 5 cm long and 2 cm wide slices.

One part of the pre-treated *U. pinnatifida* and *L. japonica* samples was used for subsequent cooking and the other part was used to prepare seasoning samples for cooking afterwards.

#### 2.2.2 Preparation of seasoning samples

The seasoning solution of *L. japonica* and *U. pinnatifida* was prepared as follows: 8 g white granulated sugar, 5 g white vinegar, 10 g mature vinegar, 5 g soy sauce, 1.5 g monosodium glutamate, 1.8 g edible salt (0.5 g edible salt for *U. pinnatifida sample*) dissolved in 100 mL of water to obtain the seasoning solution.

The pre-treated shredded *U. pinnatifida* and *L. japonica* slices were soaked in the seasoning solution at 5 ℃, *L. japonica* slices were soaked for 15 min, and the shredded *U. pinnatifida* for 2 min. Each sample was 50 g. *L. japonica* slices and shredded *U. pinnatifida* samples were immersed in 0.5 % calcium chloride for 2 min and 1.5 min respectively for subsequent cooking.

### 2.3 Cooking conditions

The pre-treated samples and the prepared seasoning samples were cooked separately in different techniques, each at 50 g.

#### 2.3.1 High pressure (HP) cooking

50 g of unseasoned pretreatment samples and 50 g of seasoned samples were taken and placed in the glass case (830 mL, Anhui Deli Daily Glass Co., Ltd., Anhui, China) in an electric pressure cooker (YBD50-90A1(B), Zhangzhou Wanlida Appliance Co., Ltd., Zhangzhou, China), the *U. pinnatifida* and *L. japonica* samples were cooked for 15 min and 20 min respectively.

#### 2.3.2 Microwaving

Shredded *U. pinnatifida* and *L. japonica* slices samples were mixed with 130 mL and 150 mL of deionized water respectively, placed in a heat-resistant glass box (KH-8676, Xitianlong Technology Development Co., Ltd., Tianjin, China) and cooked in a microwave oven (NE-1753, Panasonic Co., Ltd., Kadoma, Japan) at 1700 W for 3 min and 4 min.

#### 2.3.3 Air frying (AF)

The samples were placed in the foil wrap (ZY2242, Suzhou Spike Aluminum Foil Co., Ltd, Suzhou, China), which was folded to a size of 20 cm × 20 cm to cover the samples, and then placed in an air fryer (HD 9651, Philips (China) Investment Co. Ltd., Shenzhen, China) at 180 °C. The shredded *U. pinnatifida* and *L. japonica* slices samples were cooked for 15 min and 18 min respectively.

#### 2.3.4 Steaming

The *U. pinnatifida* and *L. japonica* samples were mixed with deionized water in a 1:1 ratio and crushed in a wall breaker (JYL-C010, Joyoung Co., Ltd, Jinan, China) for 45 s. Then, they were steamed in a steamer (QVL1526-1, Zhejiang Aishida Electric Co., Ltd., Taizhou, China) for 10 min and 15 min respectively.

#### 2.3.5 Baking

The samples were baked at 170 °C in the oven (SCC WE 101/01, Landsberg a. Lech, Germany), choosing a four-stage wind force, and the *U. pinnatifida* and *L. japonica* samples were baked for 8 min and 10 min respectively.

The cooked samples were quickly cooled down with ice water, evacuated into foil vacuum bags and stored at −20 °C until analysis.

### 2.4 Gas chromatography-ion mobility spectrometry (GC-IMS) analysis

Volatiles compounds in *U. pinnatifida* and *L. japonica* samples were analyzed by GC-IMS with modifications as described in the method of Zhang et al. (2022). Analysis was carried out using GC in combination with IMS instrumentation (Flavourspec®-G.A.S. Dortmund Company, Dortmund, Germany). 5 g of chopped sample was weighed into a 20 mL headspace flask and incubated at 60 ℃ for 15 min at 500 rpm before being measured. The injection volume was set to 500 μL and the injection needle temperature was 85 ℃. The volatile compounds were separated on an MXT-WAX column (30 m × 0.53 mm × 1 µm, RESTEK, PA, USA) at a column temperature of 60 ℃. Nitrogen was used as the detection carrier gas and the drift tube temperature was 45 ℃. The initial flow rate was 2 mL/min, which was held for two minutes and then linearly increased to 10 mL/min over 10 min and finally to 100 mL/min over the remaining 20 min. Three replicates on average were used for the final analysis.

### 2.5 Determination of E-nose

Electronic nose (PEN3, AIRSENSE, Schwerin, Germany) was used to detect odors. The test conditions were modified according to the method of Ma, Mu, and Zhou (2021). 1.0 g of sample was accurately weighed in a 10 mL vial and each sample was measured 3 times in parallel. Measurement conditions: 60 s for sensor cleaning, 10 s for auto-zeroing, 5 s for sample preparation, and 60 s for data detection.

### 2.6 Statistical analysis

Data was examined using IBM SPSS Statistics 26 (IBM Corporation, Armonk, NY, USA). On the FlavourSpec® flavor analyzer, the accompanying software was used to view the analytical spectra and qualitative and quantitative data, and the database was called for qualitative analysis of the substances. The Reporter plug-in compares the spectral differences between samples, the GalleryPlot plug-in creates fingerprint profiles and compares the volatile organic compounds differences between samples, and the Dynamic PCA plug-in performs dynamic PCA. In PCA analysis, principal component analysis was created using signal intensity to highlight the differences in volatile components. Statistical comparisons were conducted using analysis of variance (ANOVA) and Duncan's multiple range test. Significant differences were determined using SPSS software with a level of p < 0.05.

## 3 Results and discussion

The results of the identification of volatile compounds in the samples were shown in Table 1. 72 compounds were identified in *U. pinnatifida* and seasoning *U. pinnatifida*. *L. japonica* and seasoning *L. japonica* contained 70 compounds. Certain substances can generate several signals or spots depending on their concentration (e.g., due to the presence of monomers, dimers, or trimers) (Li et al., 2019).

**Table 1:** The volatile compounds identified by GC-IMS in *U. pinnatifida*, seasoning *U. pinnatifida*, *L. japonica* and seasoning *L. japonica* under different cooking techniques.

**Count**	**Compound**	**CAS#**	**Formula**	**MW**	**RI**	**Rt (*sec*)**	**Dt (a.u.)**	**Comment**
***U. pinnatifida* and seasoning *U. pinnatifida***								
1	(E)-2-nonenal	C18829566	C_9_H_16_O	140.2	1521.9	1386.542	1.40972	
2	Benzaldehyde	C100527	C_7_H_6_O	106.1	1550.0	1315.508	1.14656	
3	(E, E)-2,4-Heptadienal	C4313035	C_7_H_10_O	110.2	1519.5	1238.184	1.19724	
4	furfural	C98011	C_5_H_4_O_2_	96.1	1490.9	1169.72	1.33456	
5	(E)-2-octenal	C2548870	C_8_H_14_O	126.2	1437.8	1052.634	1.33196	Monomer
6	(E)-2-octenal	C2548870	C_8_H_14_O	126.2	1437.1	1051.308	1.821	Dimer
7	Ethyl lactate	C97643	C_5_H_10_O_3_	118.1	1353.7	890.803	1.1377	
8	6-methylhept-5-en-2-one	C110930	C_8_H_14_O	126.2	1342.5	871.21	1.17711	
9	(E)-2-heptenal	C18829555	C_7_H_12_O	112.2	1328.1	846.604	1.25741	Monomer
10	(E)-2-heptenal	C18829555	C_7_H_12_O	112.2	1327.5	845.631	1.66865	Dimer
11	3-hydroxybutan-2-one	C513860	C_4_H_8_O_2_	88.1	1290.7	786.251	1.06261	
12	(E)-2-hexenal	C6728263	C_6_H_10_O	98.1	1225.3	693.035	1.17679	Monomer
13	(E)-2-hexenal	C6728263	C_6_H_10_O	98.1	1226.6	694.76	1.51577	Dimer
14	2-pentylfuran	C3777693	C_9_H_14_O	138.2	1236.5	708.13	1.24935	
15	3-Methyl-2-butenal	C107868	C_5_H_8_O	84.1	1206.2	668.019	1.09176	
16	1-Penten-3-ol	C616251	C_5_H_10_O	86.1	1168.2	602.299	0.94461	
17	heptanal	C111717	C_7_H_14_O	114.2	1191.0	647.879	1.33527	Monomer
18	heptanal	C111717	C_7_H_14_O	114.2	1190.1	646.056	1.69741	Dimer
19	1-Pentanol	C71410	C_5_H_12_O	88.1	1257.3	737.176	1.25722	Monomer
20	1-Pentanol	C71410	C_5_H_12_O	88.1	1257.3	737.176	1.51703	Dimer
21	3-Methyl-1-butanol	C123513	C_5_H_12_O	88.1	1212.1	675.643	1.24657	Monomer
22	3-Methyl-1-butanol	C123513	C_5_H_12_O	88.1	1213.9	677.922	1.49467	Dimer
23	(E)-2-pentenal	C1576870	C_5_H_8_O	84.1	1142.1	553.997	1.10698	Monomer
24	(E)-2-pentenal	C1576870	C_5_H_8_O	84.1	1141.7	553.227	1.36095	Dimer
25	2-Methyl-1-propanol	C78831	C_4_H_10_O	74.1	1104.4	490.828	1.17417	
26	Hexanal	C66251	C_6_H_12_O	100.2	1091.6	472.552	1.26625	Monomer
27	Hexanal	C66251	C_6_H_12_O	100.2	1095.6	477.629	1.56439	Dimer
28	1-Propanol	C71238	C_3_H_8_O	60.1	1045.4	417.456	1.10905	Monomer
29	1-Propanol	C71238	C_3_H_8_O	60.1	1045.4	417.456	1.25431	Dimer
30	1-penten-3-one	C1629589	C_5_H_8_O	84.1	1034.8	405.807	1.07929	Monomer
31	1-penten-3-one	C1629589	C_5_H_8_O	84.1	1032.3	403.099	1.30729	Dimer
32	pentanal	C110623	C_5_H_10_O	86.1	996.6	366.311	1.42531	
33	2-Methylbutanal	C96173	C_5_H_10_O	86.1	925.1	315.813	1.39902	
34	Butanal	C123728	C_4_H_8_O	72.1	889.3	293.253	1.11514	Monomer
35	Butanal	C123728	C_4_H_8_O	72.1	890.3	293.856	1.28365	Dimer
36	Isopropyl acetate	C108214	C_5_H_10_O_2_	102.1	884.3	290.234	1.49076	
37	Propan-2-one	C67641	C_3_H_6_O	58.1	847.8	269.103	1.11608	
38	Propionaldehyde	C123386	C_3_H_6_O	58.1	826.8	257.632	1.14903	
39	acetaldehyde	C75070	C_2_H_4_O	44.1	742.0	216.141	0.95758	
40	Acetic acid	C64197	C_2_H_4_O_2_	60.1	1497.6	1185.351	1.05067	Monomer
41	Acetic acid	C64197	C_2_H_4_O_2_	60.1	1499.2	1189.133	1.15189	Dimer
42	1-Octen-3-ol	C3391864	C_8_H_16_O	128.2	1483.2	1152.067	1.15891	
43	ethanol	C64175	C_2_H_6_O	46.1	940.7	326.126	1.13419	
44	propyl acetate	C109604	C_5_H_10_O_2_	102.1	985.7	358.023	1.47708	
45	1-Octen-3-one	C4312996	C_8_H_14_O	126.2	1307.1	812.02	1.27724	Monomer
46	1-Octen-3-one	C4312996	C_8_H_14_O	126.2	1306.4	810.937	1.6837	Dimer
47	heptan-2-one	C110430	C_7_H_14_O	114.2	1186.4	638.458	1.26583	Monomer
48	heptan-2-one	C110430	C_7_H_14_O	114.2	1187.5	640.547	1.6248	Dimer
49	Butan-2-one	C78933	C_4_H_8_O	72.1	916.5	310.23	1.06455	Monomer
50	Butan-2-one	C78933	C_4_H_8_O	72.1	916.7	310.367	1.24719	Dimer
51	ethyl acetate	C141786	C_4_H_8_O_2_	88.1	899.4	299.416	1.33751	
52	acrolein	C107028	C_3_H_4_O	56.1	868.6	280.92	0.97957	Monomer
53	acrolein	C107028	C_3_H_4_O	56.1	867.3	280.178	1.05817	Dimer
54	methanol	C67561	CH_4_O	32.0	920.2	312.634	0.98206	
55	(Z)-hept-4-enal	C6728310	C_7_H_12_O	112.2	1248.8	725.132	1.14962	
56	dimethyl sulfide	C75183	C_2_H_6_S	62.1	807.1	247.37	0.95582	
57	Nonanal	C124196	C_9_H_18_O	142.2	1403.2	982.75	1.47642	
58	1-Hexanol	C111273	C_6_H_14_O	102.2	1365.4	911.686	1.33053	
59	octanal	C124130	C_8_H_16_O	128.2	1294.4	791.929	1.405	
60	butan-1-ol	C71363	C_4_H_10_O	74.1	1152.9	573.439	1.17997	Monomer
61	butan-1-ol	C71363	C_4_H_10_O	74.1	1153.7	574.897	1.38232	Dimer
62	4-Methyl-2-pentanone	C108101	C_6_H_12_O	100.2	1021.3	391.418	1.4804	
63	2-Methyl propanal	C78842	C_4_H_8_O	72.1	840.0	264.774	1.28053	
64	Propanoic acid	C79094	C_3_H_6_O_2_	74.1	1544.0	1539.835	1.11024	
65	(Z)-Hex-3-enol	C928961	C_6_H_12_O	100.2	1398.7	974.091	1.22668	
66	diallyl sulfide	C592881	C_6_H_10_S	114.2	1134.2	540.147	1.11787	
67	Propan-2-ol	C67630	C_3_H_8_O	60.1	924.1	315.119	1.22338	
68	3-Octanone	C106683	C_8_H_16_O	128.2	1258.4	738.685	1.30576	Monomer
69	3-Octanone	C106683	C_8_H_16_O	128.2	1257.0	736.706	1.72109	Dimer
70	(Z)-2-Penten-1-ol	C1576950	C_5_H_10_O	86.1	1332.6	854.301	0.94373	
71	ethyl heptanoate	C106309	C_9_H_18_O_2_	158.2	1336.0	860.023	1.41476	
72	1-hydroxypropan-2-one	C116096	C_3_H_6_O_2_	74.1	1308.3	814.068	1.05564	Monomer
73	1-hydroxypropan-2-one	C116096	C_3_H_6_O_2_	74.1	1305.5	809.573	1.22438	Dimer
74	cyclopentanone	C120923	C_5_H_8_O	84.1	1189.5	644.784	1.10631	
75	2-Methyl-2-pentenal	C623369	C_6_H_10_O	98.1	1159.2	585.064	1.15801	Monomer
76	2-Methyl-2-pentenal	C623369	C_6_H_10_O	98.1	1158.3	583.349	1.48772	Dimer
77	methyl acetate	C79209	C_3_H_6_O_2_	74.1	855.6	273.465	1.03284	
78	5-methylfurfural	C620020	C_6_H_6_O_2_	110.1	1551.5	1596.623	1.1297	
79	2-acetylfuran	C1192627	C_6_H_6_O_2_	110.1	1536.0	1279.271	1.11367	Monomer
80	2-acetylfuran	C1192627	C_6_H_6_O_2_	110.1	1539.4	1288.141	1.44166	Dimer
81	Methional	C3268493	C_4_H_8_OS	104.2	1472.9	1128.794	1.08862	
82	2-ethyl-3-methylpyrazine	C15707230	C_7_H_10_N_2_	122.2	1404.5	985.328	1.16893	
83	2-ethyl-6-methylpyrazine	C13925036	C_7_H_10_N_2_	122.2	1390.1	957.591	1.17713	
84	2,5-dimethylpyrazine	C123320	C_6_H_8_N_2_	108.1	1321.5	835.548	1.1209	Monomer
85	2,5-dimethylpyrazine	C123320	C_6_H_8_N_2_	108.1	1321.1	834.993	1.4958	Dimer
86	2,6-dimethylpyrazine	C108509	C_6_H_8_N_2_	108.1	1328.2	846.796	1.13982	Monomer
87	2,6-dimethylpyrazine	C108509	C_6_H_8_N_2_	108.1	1328.2	846.796	1.5314	Dimer
88	2-methylpyrazine	C109080	C_5_H_6_N_2_	94.1	1271.6	757.797	1.07381	Monomer
89	2-methylpyrazine	C109080	C_5_H_6_N_2_	94.1	1272.2	758.613	1.09619	Dimer
90	2-methylpyrazine	C109080	C_5_H_6_N_2_	94.1	1270.8	756.572	1.39379	Trimer
91	2-Ethylfuran	C3208160	C_6_H_8_O	96.1	962.1	340.916	1.04803	
93	3-Methyl butanal	C590863	C_5_H_10_O	86.1	946.4	330.051	1.40583	
94	Butan-2-ol	C78922	C_4_H_10_O	74.1	1028.1	398.568	1.14847	
95	3-methyl-2-pentanone	C565617	C_6_H_12_O	100.2	1011.8	381.571	1.17638	
96	3-pentanone	C96220	C_5_H_10_O	86.1	990.6	361.682	1.11411	Monomer
97	3-pentanone	C96220	C_5_H_10_O	86.1	990.9	361.912	1.3591	Dimer
98	3-hydroxybutan-2-one	C513860	C_4_H_8_O_2_	88.1	1289.7	784.689	1.32884	
***L. japonica* and seasoning *L. japonica***								
1	(E)-2-nonenal	C18829566	C_9_H_16_O	140.2	1521.9	1386.542	1.40972	
2	Benzaldehyde	C100527	C_7_H_6_O	106.1	1550.0	1315.508	1.14656	
3	(E, E)-2,4-Heptadienal	C4313035	C_7_H_10_O	110.2	1519.5	1238.184	1.19724	
4	furfural	C98011	C_5_H_4_O_2_	96.1	1490.9	1169.72	1.33456	
5	(E)-2-octenal	C2548870	C_8_H_14_O	126.2	1437.8	1052.634	1.33196	Monomer
6	(E)-2-octenal	C2548870	C_8_H_14_O	126.2	1437.1	1051.308	1.821	Dimer
7	Ethyl lactate	C97643	C_5_H_10_O_3_	118.1	1353.7	890.803	1.1377	
8	6-methylhept-5-en-2-one	C110930	C_8_H_14_O	126.2	1342.5	871.21	1.17711	
9	(E)-2-heptenal	C18829555	C_7_H_12_O	112.2	1328.1	846.604	1.25741	Monomer
10	(E)-2-heptenal	C18829555	C_7_H_12_O	112.2	1327.5	845.631	1.66865	Dimer
11	3-hydroxybutan-2-one	C513860	C_4_H_8_O_2_	88.1	1290.7	786.251	1.06261	
12	(E)-2-hexenal	C6728263	C_6_H_10_O	98.1	1225.3	693.035	1.17679	Monomer
13	(E)-2-hexenal	C6728263	C_6_H_10_O	98.1	1226.6	694.76	1.51577	Dimer
14	2-pentylfuran	C3777693	C_9_H_14_O	138.2	1236.5	708.13	1.24935	
15	3-Methyl-2-butenal	C107868	C_5_H_8_O	84.1	1206.2	668.019	1.09176	
16	1-Penten-3-ol	C616251	C_5_H_10_O	86.1	1168.2	602.299	0.94461	
17	heptanal	C111717	C_7_H_14_O	114.2	1191.0	647.879	1.33527	Monomer
18	heptanal	C111717	C_7_H_14_O	114.2	1190.1	646.056	1.69741	Dimer
19	1-Pentanol	C71410	C_5_H_12_O	88.1	1257.3	737.176	1.25722	Monomer
20	1-Pentanol	C71410	C_5_H_12_O	88.1	1257.3	737.176	1.51703	Dimer
21	3-Methyl-1-butanol	C123513	C_5_H_12_O	88.1	1212.1	675.643	1.24657	Monomer
22	3-Methyl-1-butanol	C123513	C_5_H_12_O	88.1	1213.9	677.922	1.49467	Dimer
23	(E)-2-pentenal	C1576870	C_5_H_8_O	84.1	1142.1	553.997	1.10698	Monomer
24	(E)-2-pentenal	C1576870	C_5_H_8_O	84.1	1141.7	553.227	1.36095	Dimer
25	2-Methyl-1-propanol	C78831	C_4_H_10_O	74.1	1104.4	490.828	1.17417	
26	Hexanal	C66251	C_6_H_12_O	100.2	1091.6	472.552	1.26625	Monomer
27	Hexanal	C66251	C_6_H_12_O	100.2	1095.6	477.629	1.56439	Dimer
28	1-Propanol	C71238	C_3_H_8_O	60.1	1045.4	417.456	1.10905	Monomer
29	1-Propanol	C71238	C_3_H_8_O	60.1	1045.4	417.456	1.25431	Dimer
30	1-penten-3-one	C1629589	C_5_H_8_O	84.1	1034.8	405.807	1.07929	Monomer
31	1-penten-3-one	C1629589	C_5_H_8_O	84.1	1032.3	403.099	1.30729	Dimer
32	3-pentanone	C96220	C_5_H_10_O	86.1	994.1	364.311	1.35882	
33	pentanal	C110623	C_5_H_10_O	86.1	996.6	366.311	1.42531	
34	2-Methylbutanal	C96173	C_5_H_10_O	86.1	925.1	315.813	1.39902	
35	Butanal	C123728	C_4_H_8_O	72.1	889.3	293.253	1.11514	Monomer
36	Butanal	C123728	C_4_H_8_O	72.1	890.3	293.856	1.28365	Dimer
37	Isopropyl acetate	C108214	C_5_H_10_O_2_	102.1	884.3	290.234	1.49076	
38	Propan-2-one	C67641	C_3_H_6_O	58.1	847.8	269.103	1.11608	
39	Propionaldehyde	C123386	C_3_H_6_O	58.1	826.8	257.632	1.14903	
40	acetaldehyde	C75070	C_2_H_4_O	44.1	742.0	216.141	0.95758	
41	Acetic acid	C64197	C_2_H_4_O_2_	60.1	1497.6	1185.351	1.05067	Monomer
42	Acetic acid	C64197	C_2_H_4_O_2_	60.1	1499.2	1189.133	1.15189	Dimer
43	1-Octen-3-ol	C3391864	C_8_H_16_O	128.2	1483.2	1152.067	1.15891	
44	ethanol	C64175	C_2_H_6_O	46.1	940.7	326.126	1.13419	
45	propyl acetate	C109604	C_5_H_10_O_2_	102.1	985.7	358.023	1.47708	
46	1-Octen-3-one	C4312996	C_8_H_14_O	126.2	1307.1	812.02	1.27724	Monomer
47	1-Octen-3-one	C4312996	C_8_H_14_O	126.2	1306.4	810.937	1.6837	Dimer
48	heptan-2-one	C110430	C_7_H_14_O	114.2	1186.4	638.458	1.26583	Monomer
49	heptan-2-one	C110430	C_7_H_14_O	114.2	1187.5	640.547	1.6248	Dimer
50	Butan-2-one	C78933	C_4_H_8_O	72.1	916.5	310.23	1.06455	Monomer
51	Butan-2-one	C78933	C_4_H_8_O	72.1	916.7	310.367	1.24719	Dimer
52	ethyl acetate	C141786	C_4_H_8_O_2_	88.1	899.4	299.416	1.33751	
53	acrolein	C107028	C_3_H_4_O	56.1	868.6	280.92	0.97957	Monomer
54	acrolein	C107028	C_3_H_4_O	56.1	867.3	280.178	1.05817	Dimer
55	methanol	C67561	CH_4_O	32.0	920.2	312.634	0.98206	
56	(Z)-hept-4-enal	C6728310	C_7_H_12_O	112.2	1248.8	725.132	1.14962	
57	dimethyl sulfide	C75183	C_2_H_6_S	62.1	807.1	247.37	0.95582	
58	Nonanal	C124196	C_9_H_18_O	142.2	1403.2	982.75	1.47642	
59	1-Hexanol	C111273	C_6_H_14_O	102.2	1365.4	911.686	1.33053	Monomer
60	1-Hexanol	C111273	C_6_H_14_O	102.2	1365.4	911.686	1.64206	Dimer
61	octanal	C124130	C_8_H_16_O	128.2	1294.4	791.929	1.405	Monomer
62	octanal	C124130	C_8_H_16_O	128.2	1295.3	793.245	1.82441	Dimer
63	3-hydroxybutan-2-one	C513860	C_4_H_8_O_2_	88.1	1289.0	783.732	1.33502	
64	butan-1-ol	C71363	C_4_H_10_O	74.1	1152.9	573.439	1.17997	Monomer
65	butan-1-ol	C71363	C_4_H_10_O	74.1	1153.7	574.897	1.38232	Dimer
66	4-methyl-2-pentanone	C108101	C_6_H_12_O	100.2	1021.3	394.849	1.17764	Monomer
67	4-Methyl-2-pentanone	C108101	C_6_H_12_O	100.2	1021.3	391.418	1.4804	Dimer
68	2-Methyl propanal	C78842	C_4_H_8_O	72.1	840.0	264.774	1.28053	
69	Propanoic acid	C79094	C_3_H_6_O_2_	74.1	1544.0	1539.835	1.11024	
70	(Z)-Hex-3-enol	C928961	C_6_H_12_O	100.2	1398.7	974.091	1.22668	
71	(E)-3-hexen-1-ol	C928972	C_6_H_12_O	100.2	1377.2	933.389	1.2382	
72	dimethyl trisulfide	C3658808	C_2_H_6_S_3_	126.3	1370.8	921.517	1.3016	
73	diallyl sulfide	C592881	C_6_H_10_S	114.2	1134.2	540.147	1.11787	
74	Propan-2-ol	C67630	C_3_H_8_O	60.1	924.1	315.119	1.22338	
75	3-Octanone	C106683	C_8_H_16_O	128.2	1258.4	738.685	1.30576	Monomer
76	3-Octanone	C106683	C_8_H_16_O	128.2	1257.0	736.706	1.72109	Dimer
77	(Z)-2-Penten-1-ol	C1576950	C_5_H_10_O	86.1	1332.6	854.301	0.94373	
78	ethyl heptanoate	C106309	C_9_H_18_O_2_	158.2	1336.0	860.023	1.41476	
79	1-hydroxypropan-2-one	C116096	C_3_H_6_O_2_	74.1	1308.3	814.068	1.05564	Monomer
80	1-hydroxypropan-2-one	C116096	C_3_H_6_O_2_	74.1	1305.5	809.573	1.22438	Dimer
81	cyclopentanone	C120923	C_5_H_8_O	84.1	1189.5	644.784	1.10631	
82	2-Methyl-2-pentenal	C623369	C_6_H_10_O	98.1	1159.2	585.064	1.15801	Monomer
83	2-Methyl-2-pentenal	C623369	C_6_H_10_O	98.1	1158.3	583.349	1.48772	Dimer
84	methyl acetate	C79209	C_3_H_6_O_2_	74.1	855.6	273.465	1.03284	
85	2-acetylfuran	C1192627	C_6_H_6_O_2_	110.1	1536.0	1279.271	1.11367	Monomer
86	2-acetylfuran	C1192627	C_6_H_6_O_2_	110.1	1539.4	1288.141	1.44166	Dimer
87	Methional	C3268493	C_4_H_8_OS	104.2	1472.9	1128.794	1.08862	
88	2-ethyl-3-methylpyrazine	C15707230	C_7_H_10_N_2_	122.2	1404.5	985.328	1.16893	Monomer
89	2-ethyl-6-methylpyrazine	C13925036	C_7_H_10_N_2_	122.2	1390.1	957.591	1.17713	Dimer
90	2,5-dimethylpyrazine	C123320	C_6_H_8_N_2_	108.1	1321.5	835.548	1.1209	Monomer
91	2,5-dimethylpyrazine	C123320	C_6_H_8_N_2_	108.1	1321.1	834.993	1.4958	Dimer
92	2,6-dimethylpyrazine	C108509	C_6_H_8_N_2_	108.1	1328.2	846.796	1.13982	Monomer
93	2,6-dimethylpyrazine	C108509	C_6_H_8_N_2_	108.1	1328.2	846.796	1.5314	Dimer
94	2-methylpyrazine	C109080	C_5_H_6_N_2_	94.1	1271.6	757.797	1.07381	Monomer
95	2-methylpyrazine	C109080	C_5_H_6_N_2_	94.1	1272.2	758.613	1.09619	Dimer
96	2-methylpyrazine	C109080	C_5_H_6_N_2_	94.1	1270.8	756.572	1.39379	Trimer
97	2-Ethylfuran	C3208160	C_6_H_8_O	96.1	962.1	340.916	1.04803	
98	3-Methyl butanal	C590863	C_5_H_10_O	86.1	946.4	330.051	1.40583	
99	5-methylfurfural	C620020	C_6_H_6_O_2_	110.1	1643.4	1583.456	1.12957	Monomer
100	5-methylfurfural	C620020	C_6_H_6_O_2_	110.1	1644.3	1586.306	1.47308	Dimer

MW: Molecular weight, RI: the retention index, Rt: the retention time, Dt: the drift time.

### 3.1 Changes in volatile flavor compounds in *U. pinnatifida* and seasoning *U. pinnatifida* in different cooking techniques

#### 3.1.1 U. pinnatifida

**Fig. 1:**
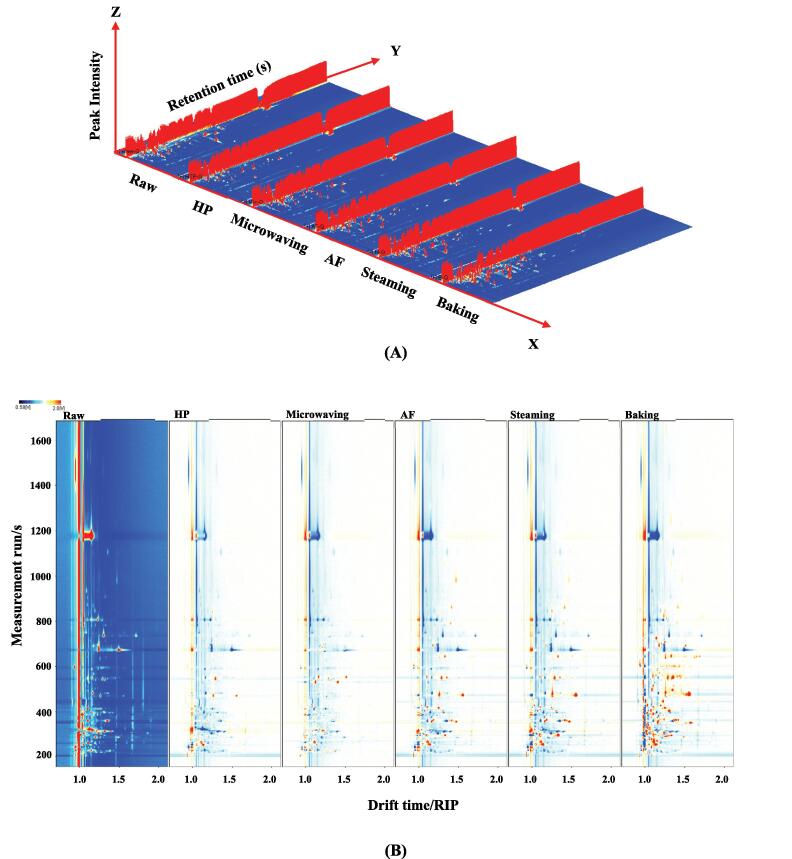

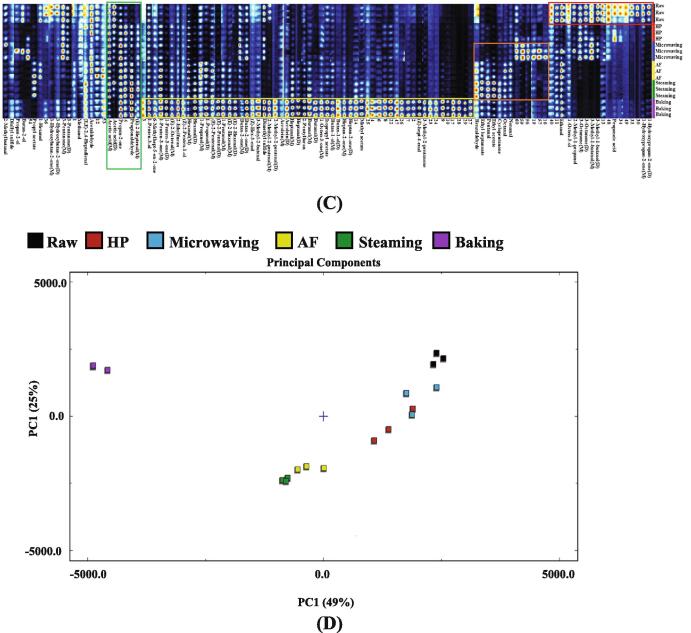
3D-topographic (A), topographic sub-traction plots (B), PCA plot (C) and fingerprint (D) of volatile compounds in *U.pinnatifida* under different cooking techniques.

GC-IMS was used to analyze the differences in volatile compounds in *U. pinnatifida* after different cooking methods. The results for *U. pinnatifida* treated with different cooking techniques were shown in the 3D topography in Fig. 1A. The drift time was displayed on the X-axis, the retention time on the Y-axis, and the ion peak intensity on the Z-axis. The fractions were tentatively identified by comparing their retention indices (RI) and mass spectrums with those of internal databases and reference compounds (Guo, Ho, et al., 2021). Each dot in the spectrum represented a volatile compound, with red representing high concentrations and white representing low concentrations. The ion peaks can distinguish between monomers, dimers and multimers of the same substance, depending on the content and nature of the substance. The differences in volatile flavor compounds in *U. pinnatifida* from the different cooking methods can be visualized in the Fig. 1A. However, in order to compare the discrepancies, the top view was obtained below due to the difficulty of observation. The difference comparison pattern for the top view of the GC-IMS 3D topography was displayed in Fig. 1B along with the normalized ion migration times and positions of the reaction ion peaks (RIP). The raw sample spectra were selected as a reference and the spectra of the reference were deducted from the other samples. The background would have been white if the volatile taste compounds in both samples were identical, with red denoting a substance's concentration being higher than the reference and blue denoting its concentration being lower. The significant increase in the number of red dots in the baked *U. pinnatifida* samples could be attributed to the increased production of volatile flavor substances due to the promotion of the Maillard reaction during the baking process (Suri, Singh, Kaur, Yadav, & Singh, 2020).

A topographic map can clearly display the cyclical changes in volatile elements. However, making an accurate assessment of the closely related compounds on the map is challenging (Yang et al., 2019). The Gallery Plot plug-in was used to automate the generation of fingerprints of all peaks to be evaluated for various cooking samples in order to better understand the variations of volatile matter in different cooking samples (Fig. 1C). Each column showed the peaks of the same volatile chemical in *U.pinnatifida* treated with various cooking techniques, and the peaks chosen from each sample were represented by a row. Individual dots indicated a volatile compound, and the shading of the color denoted the level of that volatile compound, with brighter color indicating higher levels. By comparing the dynamics of volatile compounds between samples through fingerprinting, changes in volatile compounds (increase, decrease, disappearance or fluctuation) between different cooking methods can be determined (Xuan et al., 2022). M, D and T in brackets after the substance name represented the monomer, dimer and trimer of the substance, respectively. Fig. 1C showed the differences in volatile organic chemicals between samples as well as the full information on volatile organic compounds for each sample. The substances in the red box were highest in raw *U. pinnatifida* sample and included 1-hydroxypropan-2-one, propionic acid, 3-methyl-1-butanol, 3-octanone, 2-Methyl-1-propanol, 1-octen-3-ol and ethanol, indicating that these cooking methods may have caused a loss of these flavor compounds. The substances in the yellow box were the most abundant in the baked samples and included 3-methyl-2-pentanone, (Z)-hept-4-enal, methyl acetate, heptan-2-one, butan-1-ol, isopropyl acetate, 1-octen-3-one, butanal, 2-pentylfuran, heptanal, acrolein, 2-methyl-2-pentenal, dimethyl sulfide, 3-methyl-2-butenal, (Z)-hex-3-enol, butan-2-one, (E)-2-hexenal, 1-pentanol, (E)-2-pentenal, 1-propanol, hexanal, *cis*-2-penten-1-ol, 2-ethylfuran, *trans*-2-octenal, 1-penten-3-one, 6-methylhept-5-en-2-one and 1-penten-3-ol. Baking may have provided gentler and more sustained heating, which helped more kinds of compounds to react thermally, leading to richer flavors. The substances in the orange box were highest in one type of *U. pinnatifida*, with nonanal and octanal being the most abundant in the AF cooking. They were aliphatic aldehydes, which usually with aromas of citrus, orange, fat, or greasy. Air frying was cooking by strong convection air flow. The air movement may result in more oxygen being involved in chemical reactions in the ingredients, including oxidation, which promoted the production of nonanal and octanal. Cyclopentanone, ethyl acetate, pentanal, ethyl acetate and benzaldehyde were present in the highest amounts in the steamed samples. Steaming was usually done at relatively high but mild temperatures. This may prompt some chemical reactions that make it easier for some volatile compounds such as cyclopentanone and ethyl acetate to evaporate from the ingredients. The compounds in the green box were the least abundant in one variety of *U. pinnatifida*, with (E)-2-heptenal, propionaldehyde and propan-2-one being the least abundant in raw sample, acetic acid was the least abundant in baking.

Fig. 1D showed the principal component analysis (PCA) of *U. pinnatifida* in different cooking methods. And all the data included in PCA came from the same batch of tests. PCA is a multivariable statistic method for assessing correlations between various variables. It is a multidimensional data analyzer tool for analyzing multidimensional data sets with quantifiable variables (An, Liu, Hao, Wang, & Tang, 2020). The original variables, which contain 3D data concerning retention time, drift time, and ion signal intensity, are used to create a number of principal components (as variables, mainly as observations) (Wang et al., 2019). Signal intensities are used to emphasize changes in volatile components, which may be seen between samples, in order to synthesize the issue. Additionally, the graph can visualize the differences between the samples, samples with overlap or closeness have scent profiles that are comparable. On the other hand, differences become more obvious the further apart the samples are (Hernandez et al., 2016, Song et al., 2020). In general, the PCA model was regarded as the preferred separation model when the total contribution of PC1 and PC2 reached 60 % (Wu et al., 2015). As seen in Fig. 1D, the contribution of PC1 was 49 % and PC2 was 25 %. The accumulated contribution was 74 %, which was adequate to interpret how comparable the various samples were. Fig. 1D revealed that the difference between samples from the baking group and the other samples was significant.

#### 3.1.2 Seasoning *U. pinnatifida*

**Fig. 2:**
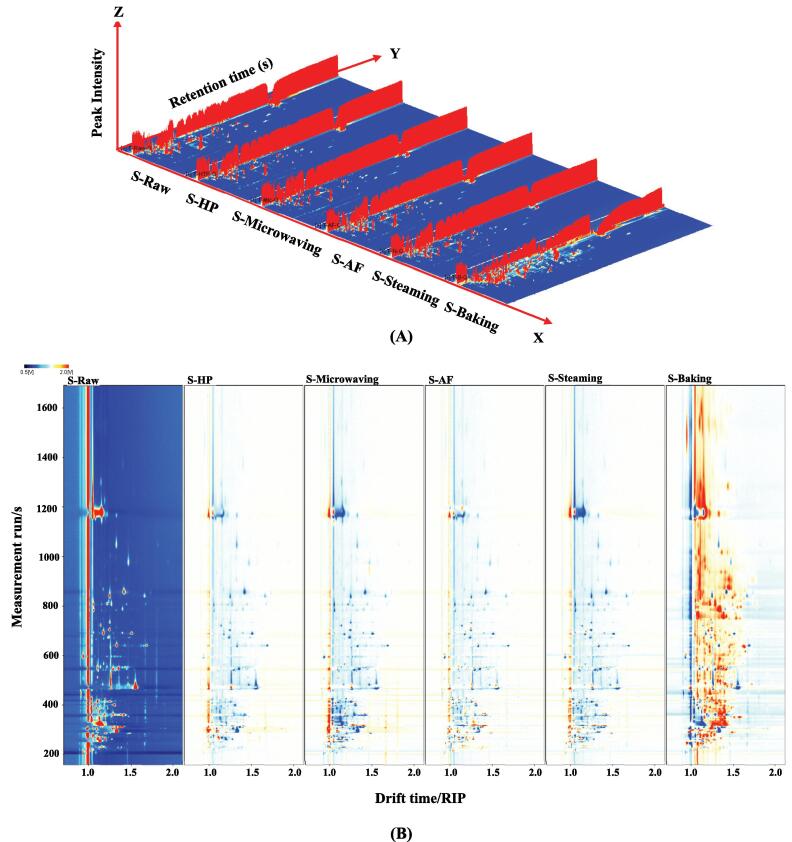

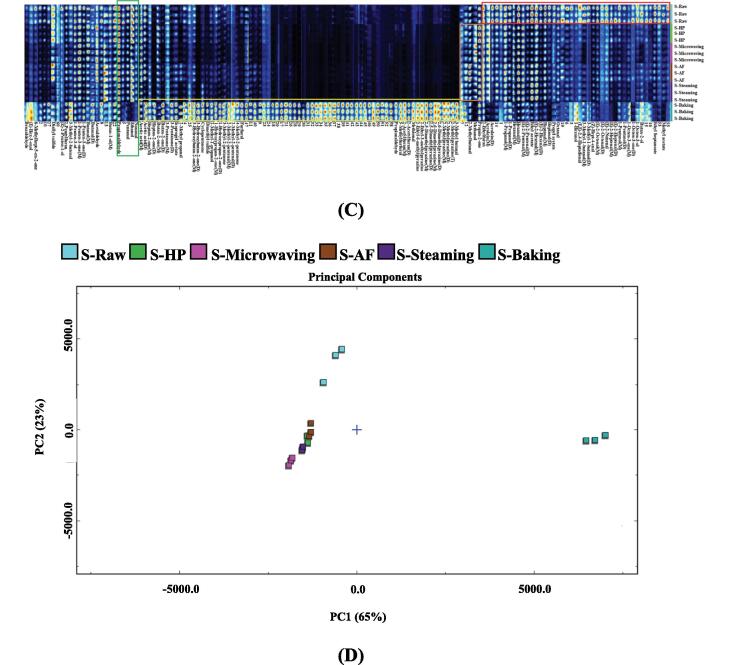
3D-topographic (A), topographic sub-traction plots (B), PCA plot (C) and fingerprint (D) of volatile compounds in seasoning *U.pinnatifida* under different cooking techniques.

The flavor compounds of seasoned *U. pinnatifida* treated with different cooking techniques were characterized in Fig. 2. Similar to the *U. pinnatifida* samples, the 3D plots (Fig. 2A) of the seasoned *U. pinnatifida* samples did not show the differences between the groups in great detail, so in order to compare the differences between the groups in more detail and visually, the reference spectra deducted from the other samples to obtain the difference comparison pattern, as shown in Fig. 2B. It can be seen that the seasoned *U. pinnatifida* still differed significantly from the control group in the baking group after treatment with different cooking techniques. As shown in Fig. 2C, the substances in the red box in the fingerprint profile were the most abundant in raw seasoning *U. pinnatifida* sample and included methyl acetate, ethyl heptanoate, butan-2-ol, 1-octen-3-ol, 1-octen-3-one, 1-pentanol, (E)-2-heptenal, (E)-2-nonenal, (E)-2-octenal, (Z)-hept-4-enal, 3-methy-1-butanol, (E,E)-2,4-heptadienal, 1-hexanol, octanal, propyl acetate, heptanal, (E)-2-hexenal, ethyl acetate, (E)-2-pentenal, hexanal, 1-propanol, acrolein and 2-ethylfuran. The substances in the yellow box were highest in baking samples of seasoned *U. pinnatifida* and included 3-methyl butanal, 2-methylpyrazine, 2,6-dimethylpyrazine, 2,5-dimethylpyrazine, 2-ethyl-3-methylpyrazine, 2-ethyl-6-methylpyrazine, methional, 2-acetylfuran, 5-methylfurfural, propionic acid, furfural, 4-methyl-2-pentanone, 2-methyl-2 pentenal, 3-methyl-2-pentanone, 1-hydroxypropan-2-one, 2-Methyl-2-pentenal, dimethyl sulfide, cyclopentanone, 3-hydroxybutan-2-one, 2-Methyl propanal, isopropyl acetate, 3-pentanone, butan-2-one, heptan-2-one and acetic acid. The substances in the orange box were highest in one type of seasoned samples, with Propan-2-one in the HP sample, methanol in the microwaving and 2-methylbutanal in the AF group. Flavor compound substances in the green box, such as nonanal, ethanol, pentanal and propionaldehyde, were at their lowest levels under the baking treatment.

The PCA results made it quite evident that that the seasoned *U. pinnatifida* samples treated with different cooking techniques could be distinguished in the distribution map in a relatively independent space. In Fig. 2D, the distribution of the HP, microwaving and AF treated seasoned samples was close to each other, indicating little change in flavor substances in the samples, while there was a significant deviation in the baking samples, indicating a significant difference in volatile flavor compounds in the baking samples.

### 3.2 Changes in volatile flavor compounds in *L. japonica* and seasoning *L. japonica* in different cooking techniques

#### 3.2.1 L. japonica

**Fig. 3:**
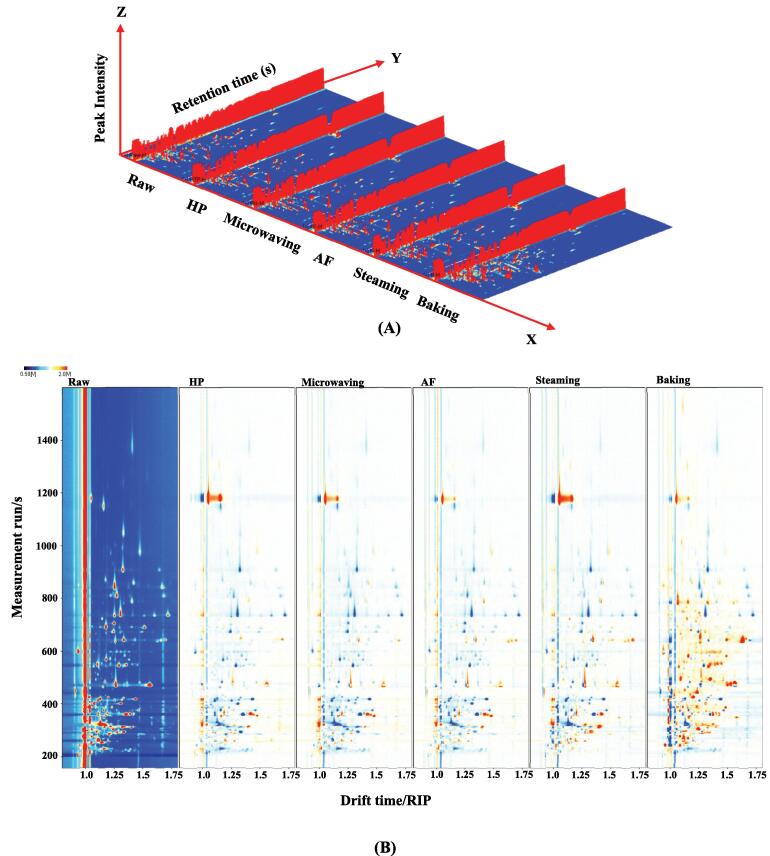

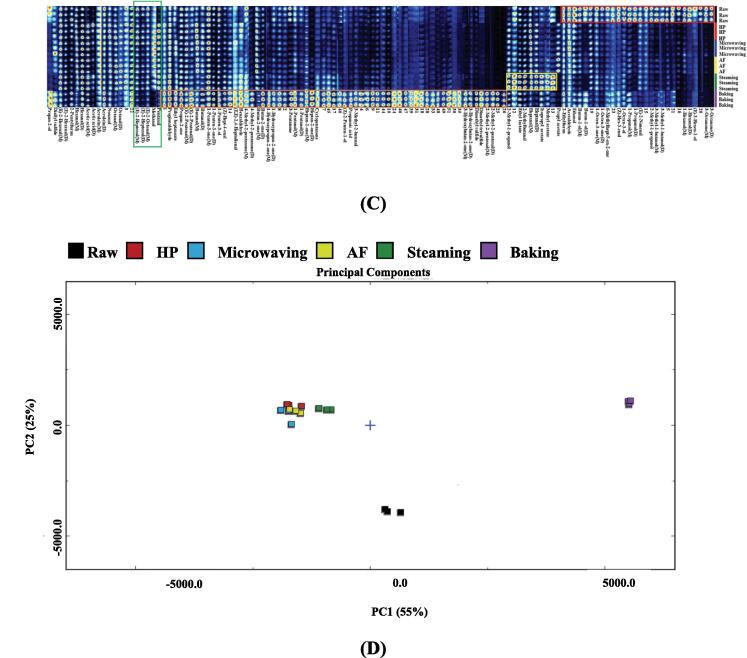
3D-topographic (A), topographic sub-traction plots (B), PCA plot (C) and fingerprint (D) of volatile compounds in *L. japonica* under different cooking techniques.

The 3D topography and topographic sub-traction plots of GC-IMS of volatile organic compounds in *L. japonica* treated with different cooking methods was shown in Fig. 3A and Fig. 3B. The graph showed that the baked samples were significantly different from the control group compared to the other cooking groups, which was consistent with the PCA analysis (Fig. 3D). Nevertheless, the samples in HP, microwaving, AF and steaming groups were relatively similar in terms of volatile organic compounds. The fingerprint map (Fig. 3C) showed that the substances in the red box were the most abundant in the control group, including 3-octanone, (E)-3-hexen-1-ol, 1-hexanol, 3-methyl-1-butanol, 2-methyl-1-propanol, (E)-2-nonenal, 1-propanol, 1-octen-3-ol, (Z)-Hex-3-enol, 6-methylhept-5-en-2-one, 1-octen-3-one, butanol-1-ol, ethanol, acetaldehyde and 2-ethylfuran. And propyl acetate, to the left of the red box, was the most abundant in the AF sample. The substances in the yellow boxes were the most abundant in the steamed samples and included methyl acetate, isopropyl acetate, heptanal, 2-methylbutanal, ethyl lactate and 2-methyl propanal. The substances in the orange frame with the highest content in the baked *L. japonica* include 2-methyl-2-pentenal, dimethyl trisulphide, diallyl sulfide, 3-hydroxybutan-2-one, 3-methyl-2-butenal, propionic acid, (Z)-2-Penten-1-ol, cyclopentanone, heptan-2-one, 1-pentanol, 3-pentanone, 1-hydroxypropan-2-one, Butan-2-one, 4-methyl-2-pentanone, benzaldehyde, (E,E)-2,4-heptadienal, (Z)-hept-4-enal, 1-penten-3-ol, 1-penten-3-one, butanal, (E)-2-pentenal, propan-2-one, ethyl heptanoate and propionaldehyde. The substances in the green box were present at the lowest levels in one of the *L. japonica* samples, with g pentanal being the lowest in the control group. Methanol, (E)-2-octenal and (E)-2-heptenal were present at the lowest levels in the baked samples.

#### 3.2.2 Seasoning *L. japonica*

**Fig. 4:**
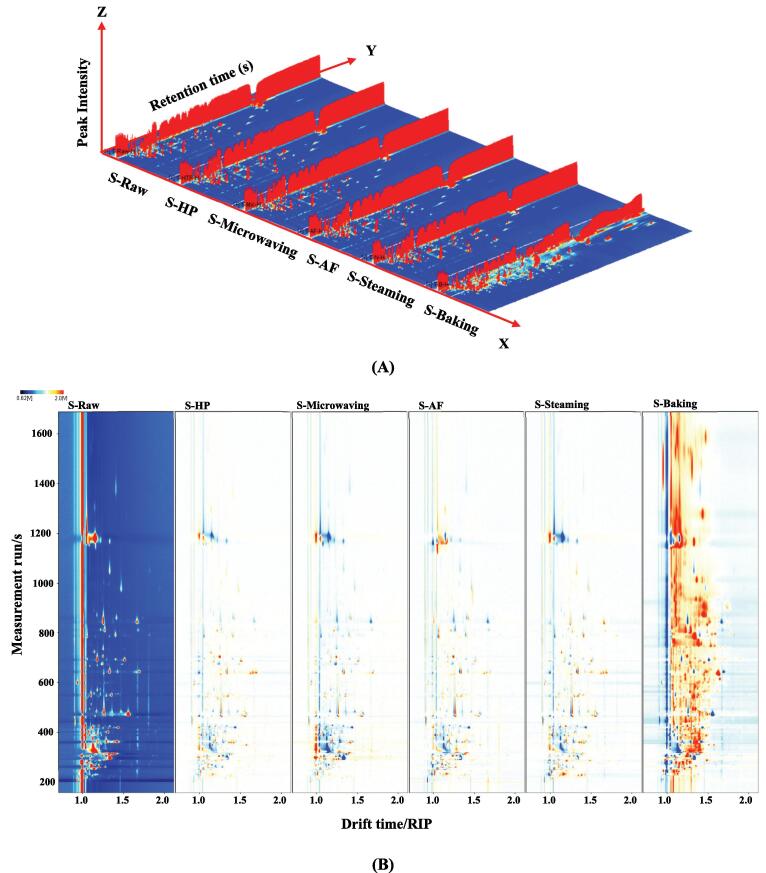

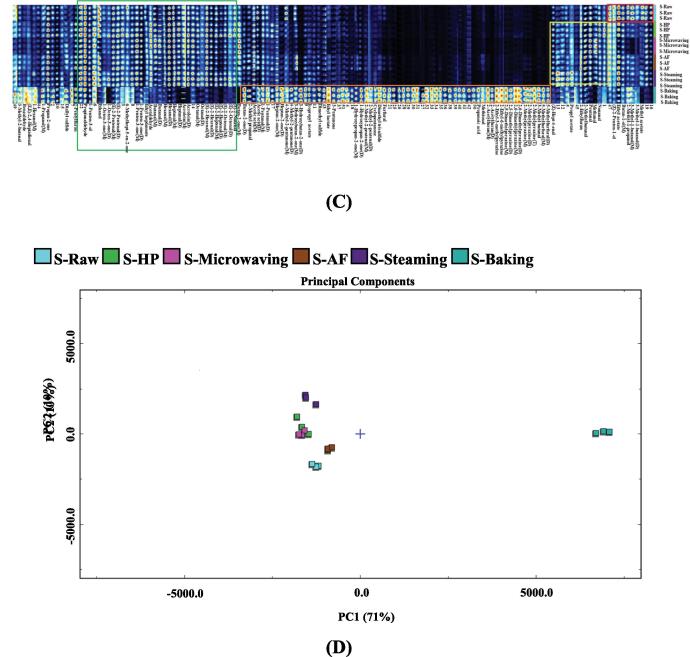
3D-topographic (A), topographic sub-traction plots (B), PCA plot (C) and fingerprint (D) of volatile compounds in seasoning *L. japonica* under different cooking techniques.

Similar to the previous results, the topographic sub-traction plots in Fig. 4B revealed significant differences between the baked samples and controls in the seasoning *L. japonica* treated with different cooking techniques. The PCA analysis distance (Fig. 4D) of other cooking methods was similar, i.e., the volatile flavor substances were similar. Detailed information was shown in the fingerprint (Fig. 4C). The substances in the red box were the most highly concentrated in seasoned *L. japonica*, including methyl acetate, 3-methyl-1-butanol, 2-Methyl-1-propanol, butan-1-ol, ethyl acetate and (Z)-2-Penten-1-ol. The highest levels of substances in the orange frame were found in baked seasoning *L. japonica* and included 5-methylfurfural, 3-Methyl butanal, 2-methylpyrazine, 2,6-dimethylpyrazine, 2,5-dimethylpyrazine, 2-ethyl-6-methylpyrazine, 2-ethyl-3-methylpyrazine, 2-acetylfuran, methional, propionic acid, furfural, dimethyl trisulfide, cyclopentanone, 2-methyl-2-pentenal, 1-hydroxypropan-2-one, 3-pentanone, ethyl lactate, dimethyl sulfide, isopropyl acetate, 3-hydroxybutan-2-one, 4-methyl-2-pentanone, heptan-2-one, 1-pentanol, acetic acid, 2-methyl propanal and butan-2-one. As shown in the yellow box, nonanal was highest in HP, methanol and pentanal in microwaved seasoned samples, 2-methylbutanal and 2-ethylfuran in AF, and propyl acetate in steamed samples. The substances in the green box were the lowest in baking and include (E)-2-nonenal, (E)-2-octenal, (E)-2-heptenal, (E)-2-hexenal, octanal, acrolein, heptanal, hexanal, acetaldehyde, Butanal, ethyl heptanoate, 1-penten-3-one, 6-methylhept-5-en-2-one, (E)-2-pentenal, 1-octen-3-one, ethanol, 1-penten-3-ol and propionaldehyde.

### 3.3 Differences in aroma distribution between samples analyzed using E-nose

**Fig. 5:**
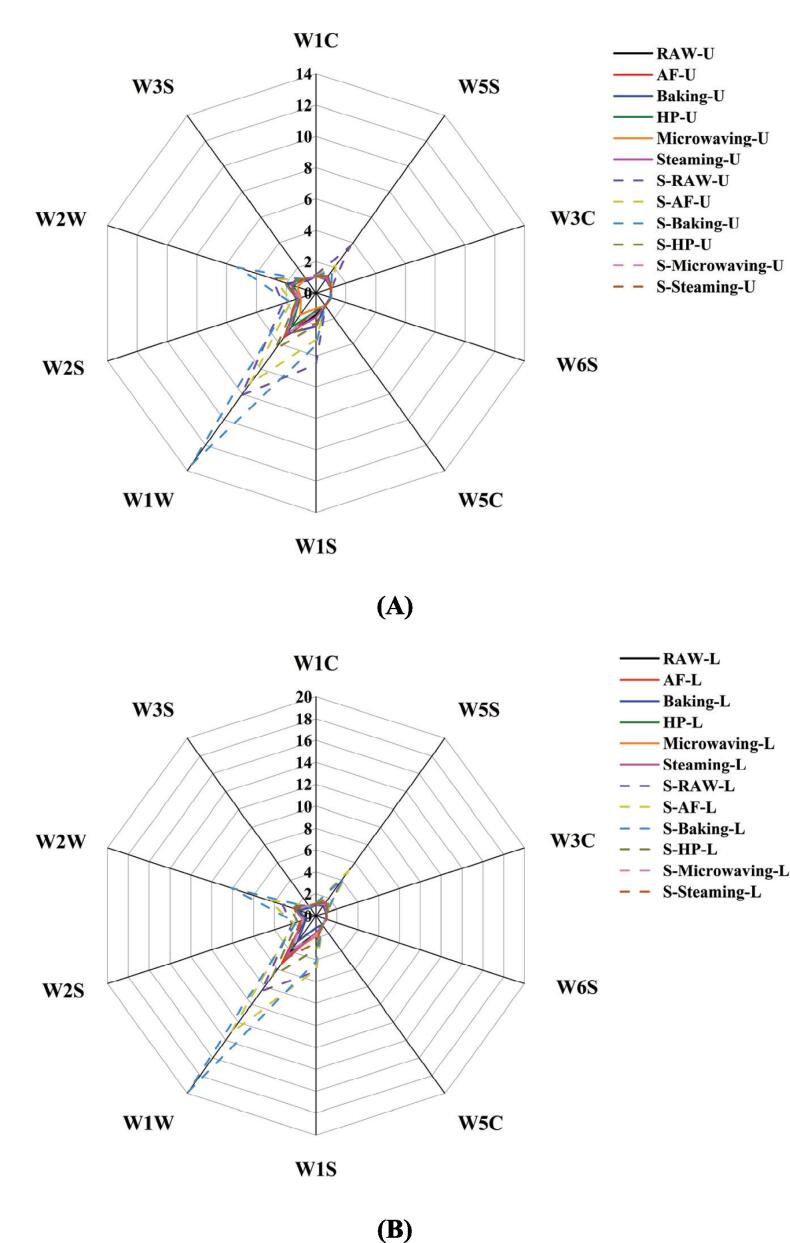
Radar map based on the E-nose data for *U. pinnatifida*, seasoning *U. pinnatifida L. japonica*, and seasoning *L. japonica*.

To comprehend aroma properties and volatile chemicals, the combined analysis of GC-IMS and E-nose data was essential (X. H. Huang et al., 2019, Lan et al., 2021, Niu et al., 2019). The E-nose simulated the human olfactory system and was widely used to identify odors (Baietto and Wilson, 2015, Xu et al., 2020). In order to accurately assess the aroma spectrum of *U. pinnatifida* and *L. japonica*, we used the E-nose, which was frequently used for aroma identification and discrimination and which provided an overall spectrum of volatile compounds but not specific information on the composition and amount of volatile compounds (Wu et al., 2021). As shown in Fig. 5A for the E-nose radar map of *U. pinnatifida* and seasoning *U. pinnatifida* under different cooking techniques, there were differences in sensor response values between the samples, indicating different aroma distributions between them. The response values of *U. pinnatifida* treated with different cooking techniques basically overlapped, which indicated that the cooking techniques had no significant effect on the aroma distribution in the *U. pinnatifida*. The response values of W1W (mainly sensitive to terpenoids and organic sulfides), W2W (mainly sensitive to aromatic compounds and organic sulfides) increased significantly in the seasoning *U. pinnatifida*, while the other response values increased slightly (Junxing et al., 2022). Among them, the response values of baked seasoning *U. pinnatifida* were significantly higher than the other groups, which was consistent with the results of GC-IMS (Fig. 2B), indicating baking produced richer flavor substances. The *L. japonica* samples were also the same as the *U. pinnatifida* samples before seasoning, with a dense distribution among the cooking groups and no significant changes as in Fig. 5B. Similarly, the response values of W1W and W2W were significantly increased in the E-nose for the seasoning *L. japonica* samples. These results may be related to the substances in the added seasoning.

## 4 Conclusion

The combination of GC-IMS and electronic nose was an effective analytical method for volatile spectra and flavors. In this study, GC-IMS was used to determine and characterize volatile compounds in seasoning solution treatment and different cooking techniques. 72 characteristic volatile compounds were detected in *U. pinnatifida* before and after seasoning. And a total of 70 compounds were detected in seasoned and unseasoned *L. japonica*. Although the GC-IMS results showed that the flavor compounds of the baked samples differed significantly from the other cooking techniques, the E-nose analysis showed that overall the treatments of the different cooking techniques had little effect on the flavor compounds in *U. pinnatifida* and *L. japonica*. In contrast, the seasoned edible brown seaweeds samples had a better flavor profile relative to the unseasoned samples. The different volatile flavor components of brown seaweeds processed by seasoning solution and different cooking methods not only provide a reference for the commercial processing of brown seaweeds, but also provide a reference for different consumers to choose suitable cooking methods, so that they can find a cooking method that better meets their own tastes and needs. However, this study has some limitations. The effects of different cooking conditions (e.g. cooking time or temperature) and seasoning solution types need to be explored more in the future.

## CRediT authorship contribution statement

**Shan Jiang:** Writing – original draft, Formal analysis, Data curation, Conceptualization. **Pengfei Jiang:** Validation, Software. **Dingding Feng:** Conceptualization. **Meiran Jin:** Validation, Conceptualization. **Hang Qi:** Writing – review & editing, Software, Resources, Methodology, Conceptualization.

## Declaration of competing interest

The authors declare that they have no known competing financial interests or personal relationships that could have appeared to influence the work reported in this paper.

## Data Availability

Data will be made available on request.
